# Urethral Instillation of Povidone-Iodine Reduces Post-Cystoscopy Urinary Tract Infection in Males: A Randomized Controlled Trial

**DOI:** 10.1038/s41598-020-60522-4

**Published:** 2020-02-27

**Authors:** Rishi Nayyar, Rohit Dadhwal, Arti Kapil, Ravindra Mohan Pandey, PremNath Dogra

**Affiliations:** 1 Department of Urology, AIIMS, New Delhi 110029 New Delhi, India; 2 Department of Microbiology, AIIMS, New Delhi 110029 New Delhi, India; 3 Department of Biostatistics, AIIMS, New Delhi 110029 New Delhi, India

**Keywords:** Bacterial infection, Urinary tract infection, Urological manifestations, Urethra, Urological manifestations

## Abstract

Office cystoscopy may be associated with urinary tract infection (UTI) in up to 10–20% of patients. Current practice of surgical part preparation in males with povidone-iodine excludes distal urethra in males, leaving a possibility for resident intra-urethral flora to cause post-procedural UTI. We designed this randomized study to assess whether additional cleaning of distal urethra with povidone-iodine solution can help reduce post-procedural incidence of UTIs in this setting. Additionally, urethral swab culture was done in the entire cohort to identify the prevalent microflora in the distal male urethra and to evaluate its role in causation of post-procedural UTI. Using a specialized urethral swab culture methodology, 85% males demonstrated some bacteria and 16% showed common uro-pathogens. 28 (14.5%) cases had post-procedure culture positive UTI. The incidence of UTI in control group (22%) was significantly more than the intervention group (7%) (p value <0.007). This result strongly supports inclusion of distal urethral irrigation with povidone-iodine in males before office cystoscopy, even when pre-procedure mid-stream urine culture is sterile.

## Introduction

Post-procedural genito-urinary tract infection (UTI) is a frequent occurrence in the clinical setting with associated morbidity, cost of treatment and possible long term sequelae^1^. Even though the magnitude of problem is huge given the large numbers of patients undergoing cystoscopy for various reasons, the role of peri-procedural prophylactic antibiotics is still not clear. The AUA (American Urological Association) Best Practice statement recommends antibacterial prophylaxis before cystoscopy in patients at risk for UTI^2^. Advanced age, smoking, anatomical anomalies, steroid use, decreased immune function and urethral catheters have been described as risk factors for UTI. All these factors either reduce the host immunity to defend against plausible invasion of microorganisms or enhance the colonization in the urethra. Even though all precautions are taken to maintain sterility of the instruments, current standard preparation of the surgical site for cystoscopy covers only the external aspect of male genitalia. However, resident flora also does reside inside the urethral lumen, particularly the distal portion and it is not clear from the available literature whether or not including the distal urethra in the surgical part preparation protocol can help reduce the incidence of post-procedural UTI after cystoscopy.

The terminal portion of the male urethra is covered with stratified squamous epithelium and colonization of the urogenital tract by several microorganisms does occur after birth. Many factors control this phenomenon, including keratinization and mucus in the epithelial surface, bacterial adherence, microbial interaction, antimicrobial substances phagocytosis, and humoral and cellular immunities^3^. With a few rare exceptions, urinary tract infection is caused by resident indigenous organisms, in particular those of the bowel and distal urethral flora, and diagnosis is bedevilled by the need to discriminate between contamination and infection of urine specimens. However, in the setting of an invasive surgical procedure like cystoscopy with associated abrasion/stretching of the mucosal surface and additional use of irrigation fluid under significant pressure, these otherwise non-detrimental resident florae may become clinically relevant and cause actual invasion manifesting as various forms of genitourinary infection, depending upon the site of lodgement.

The microbiota of the male reproductive tract is itself poorly described in literature. The penis provides a distinct anatomical environment in the urethra. Some bacteria transferred during sexual activity and some present as commensals may be a cause for substantial morbidity^4^. In addition, the coronal sulcus and distal urethra of healthy men at least episodically supports bacterial communities^5–8^.

In this background, the study was designed to assess whether additional cleaning of distal urethra with povidone-iodine solution can help reduce post-procedural incidence of UTIs in men scheduled to undergo lower urinary tract endoscopy in our tertiary care centre. We also planned to identify the prevalent intra-urethral microflora in all these men and to correlate the type of bacterial growths with the occurrence of post-procedural UTI in this setting.

## Materials and Methods

A double blind randomized controlled study was done in the Department of Urology in collaboration with the Department of Microbiology. The trial was duly registered with the National Trial Registry, India (CTRI/2019/08/020623) [Registered on 07/08/2019], and approved by the Institutional review board and ethical committee, All India Institute of Medical Sciences, New Delhi (Ref No. IECPG-561/20.12.2017,RT-9/31.01.2018). Entire methodology was designed and carried out in accordance with the contemporary urology guidelines^2^ and local regulations. An informed written consent was obtained from all prospective participants after explanation of the methods in local vernacular language both written and descriptive. All consecutive male patients of 15–75 year age group undergoing office cystoscopy with no significant [>10^5^colony forming units (cfu)/ml) growth on routine bacterial urine culture (done within 1 week of scheduled procedure) were considered for inclusion. All urine cultures were done in our microbiology laboratory. Exclusion criteria were any known immunodeficiency or prolonged steroid use; Indwelling catheter for more than 1 week; Cystoscopy for stone manipulation, genitourinary tuberculosis, radiation cystitis, or gross hematuria; Any other unrecognized pathology requiring significant intervention during or after cystoscopy; Emergency cystoscopy procedure; Bladder or urethral perforation during endoscopy, and; Catheter placement for more than 3 days after cystoscopy. We did not exclude cases with controlled diabetes mellitus (HbA1c <6.5 gm%), previous history of UTI’s or recent use of antibiotics, but none of our patients had antibiotic within the last week for any indication.

No peri-procedural prophylactic antibiotics were used in concurrence with current AUA best practice guidelines^2^. Medical co-morbidities of all patients were noted and optimized before endoscopy in consultation with relevant departments as needed.

All men were instructed to trim the pubic hair at home by themselves one night beforehand and have a regular bath on the morning of cystoscopy. Shaving was disallowed and no bowel preparation was prescribed. The endoscopy was done as per standard practice using rigid 17 F cystoscope under local anesthesia with 2% lignocaine gel. Sterility of all equipment was maintained throughout the procedure as per institutional protocols. The instruments were disinfected by immersion in 2% activated glutaraldehyde (Cidex^®^, Johnson & Johnson) 20 minutes before each session as recommended by AUA. Same trained technician and nursing staff was involved in the entire course of study.

Part preparation was done with povidone-iodine (5% w/v) solution from umbilicus to mid-thighs and disposable sterile draping. The glans and prepuce were washed with saline and then a culture swab sample was collected from distal urethra (the navicular fossa and penile urethra).

As a prelude to this study, a pilot study was conducted at our centre to standardize the procedure of bacterial culture. In the first 10 cases, no growth of organism was obtained when a swab culture was taken from distal urethra and incubated using standard technique on blood agar aerobic medium for 24 hours. The technique was thus modified in the next 60 cases and also followed during the entire study period. After aerobic incubation for first 24 hours in 1% brain heart infusion broth (BHI) at 37 °C, its one micro loop was inoculated on blood and MacConky’s agar mediums as anaerobic incubation for further 24 hours at 37 °C. The cultures were read manually. Only the most prevalent growth was noted for analytical purposes. We were able to demonstrate positive swab cultures in 43 out of 60 cases with this modified technique. A systematic alternating 1:1 allocation to control and intervention groups (as defined later) was done in this pilot project. 12 cases developed UTI post-cystoscopy, 3 in intervention and 9 in control groups. The results from the pilot study were used to determine the sample size as per the objectives of the study, yielding a requirement of 81 cases to describe the urethral commensal profile with absolute precision of 10%. With power of 90% and type I error (α error) 5%, a relative risk ratio of 0.333 basis points and baseline risk of 30% in control group, the minimum estimated sample size was 92 in each group.

Randomization was done after collecting the urethral swab culture using computer generated simple random table with 1:1 allocation. Allocation concealment was done using opaque envelopes picked up by patient himself before proceeding for cystoscopy. After cleaning and draping, urethral swab culture was taken. Envelope was then opened by the nursing/technical staff. The distal urethral surgical preparation was also done in the intervention group by irrigating it with 10 ml povidone-iodine 2% w/v solution for 2 min before the start of cystoscopy. The clinical assessor and interpreter of cultures were both blinded to each other.

The primary end-point was the incidence of post-procedural UTI among the two groups. Post-procedure mid-stream clean catch urine culture and urine routine microscopy specimen was obtained whenever the patient presented with new symptoms. Otherwise cultures were obtained from every patient at 2, 7 and 30 days to assess for bacteriuria. Cultures upto 2^nd^ day, 2–7^th^ day and 7–30^th^ day were thus clubbed into three groups. Patients were instructed on how to prevent contamination when collecting the mid-stream urine specimen. They were also advised to expect some irritation for the first few hours after cystoscopy which may continue for 48 hours. Instructions were given to report immediately in case of persistent symptoms, fever or anynew symptom. Blood culture was planned in patients showing signs and symptoms of sepsis. All cases were followed for 30 days after the procedure for the development of any symptomatic UTI. For the study purposes, UTI was defined as any new onset genitourinary symptom and/or significant bacteriuria (≥10^5^cfu) on urine examination. Appropriate therapeutic antibiotics and supportive measures were given if urinary symptoms persisted for more than 48 hours in the presence of significant bacteriuria, or in presence of clinical features of sepsis, prostatitis or funiculo-epididymo-orchitis.

Data were analyzed using statistical software (Stata 14). Chi–square/ Fisher exact test was used for categorical data and t-test for continuous data set to check the statistical significance of the data differences. p- value < 0.05 was considered as statistically significant.

## Results

**Figure 1:**
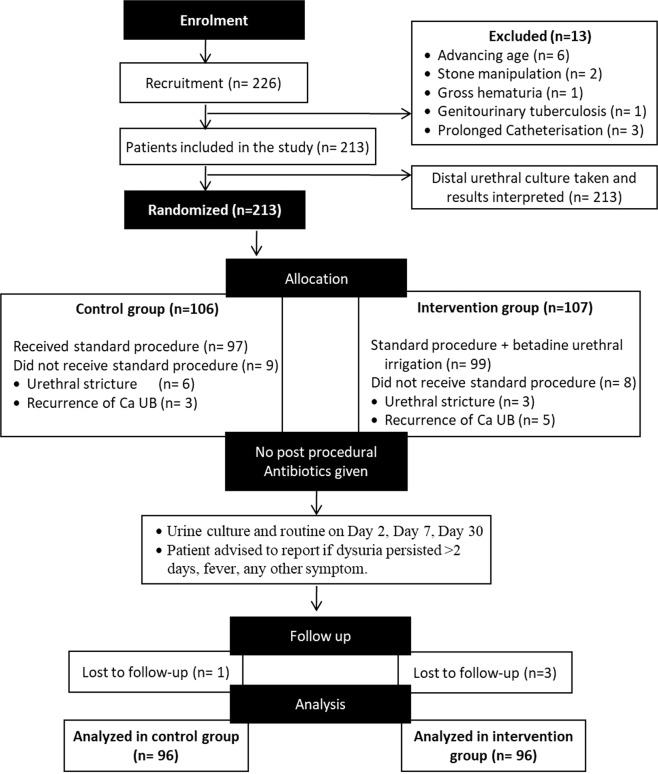
Consort diagram for the study.

A total of 226 patients from January 2018 till January 2019, who fulfilled the inclusion criterion were recruited in the study. 13 cases were excluded for various reasons. Final analysis was done in 192 patients with each group comprising of 96 patients as given in Fig. 1. Meanage of the patients was 56.5 (19–74) years and average time taken to do the cystoscopy was 9.67 (5–16) minutes.

### Resident floral profile

188 patients out of 192 showed growth of bacteria (Table 1). Most common species were coagulase negative *Staphylococcus* in 98 (51%) and *Enterococcus* in 35 (18.2%) patients. Aerobic spore bearers which depicted contamination were seen in 25 (13%) patients. Culture was sterile in 4 (2%) subjects.

**Table 1:** Resident floral profile of swab cultures from distal urethra of men undergoing office cystoscopy.

Swab culture	Number of cases
*Staphylococcus species*	**98**
*Hemolyticus*	41
*Epidermidis*	42
*Aureus*	10
*Hominis*	**5**
*Enterococcus species*	**35**
*Faecalis*	29
*Avium*	3
*Cloacae*	3
*Escherichia Coli*	**13**
*Streptococcus species*	**6**
*Klebsiellapneumoniae*	**3**
*Morganella morganii*	**3**
*Proteus Mirabilis*	**3**
*Pseudomonas aeruginosa*	**2**
*Aerobic spore bearers (Contaminants)*	**25**
Sterile	**4**
Total	**192**

### Patient characteristics

Both groups were similar with respect to age, mean hemoglobin, co-morbidities, length of cystoscopy procedure, indication for cystoscopy and commensal bacterial growth on urethral swab (Table 2). No patient in either study group had post-cystoscopy catheter placement. Those cases which had catheter placement after urethrotomy got excluded on account of an additional significant procedure and catheter placement for 5 days.

**Table 2:** Demographic description and comparison of baseline parameters in the two groups. Independent t-test (continuous variables) or Fischer’s exact test (categorical variables) was used to test for the significance of differences of means.

Category	Control Group	Intervention Group	P value
Mean age (years)	58.2 ± 11.6	55.0 ± 11.5	0.057
Mean hemoglobin (g/dl)	12.3 ± 1.5	12.0 ± 1.3	0.056
Mean length of cystoscopy (min)	9.47 ± 2.0	9.8 ± 2.1	0.193
Reason for cystoscopy			
a) Follow up for bladder cancer	94	96	0.497
b) Follow up for upper tract urothelial cancer	2	0	
Co-morbidities			
Hypothyroidism	2	5	0.44
Coronary artery disease	7	6	0.74
Hypertension	8	9	0.79
Benign prostatic obstruction	7	6	0.74
Diabetes mellitus	10	12	0.82
Swab culture			
a) Any bacterial growth except aerobic spore bearers	84	81	0.533
b) Uro-pathogenic bacterial growth (excluding aerobic spore bearers and staphylococci species)	34	31	0.76

### Episodes of UTI’s

A total of 28/192 (14.5%) cases of post-procedure culture positive UTI were encountered in the entire cohort. All these cases were managed with antibiotics on the out-patient basis not requiring any admission or ancillary procedures and thus considered as Grade II under the Clavien-Dindo classification system. The incidence of UTI in control group 21/96 (22%, 95% Confidence Interval: 13.6–30.1) was statistically significantly more than the intervention group 7/96 (7%, 95% Confidence Interval: 2.08–12.4) (p value < 0.007) using the Chi- square test (Table 3). The risk for developing UTI was reduced by more than two-thirds. Episodes of febrile UTI’s were also seen more commonly in control group; 10 (5.2%, 95% Confidence Interval: 4.3–16.5) as compared to 3 (1.5%, 95% Confidence Interval: 0.35–6.61) in intervention group though not reaching statistical significance (p value = 0.081).

**Table 3:** Comparison of episodes of UTI’s between the two groups.

Episodes of UTI			
UTI	CONTROL	INTERVENTION	TOTAL
NO	75 (78%)	89 (92.7%)	164 (85.4)
YES	21 (22%)	7 (7.2%)	28 (14.6%)
TOTAL	96 (100%)	96 (100%)	192 (100%)

### Organisms causing UTI and correlation with swab culture

Post-procedure UTI was caused most commonly by *E coli* in 18 (64%) cases followed by *Pseudomonas* and *Klebsiella* in 3 (10.7%) cases each. *Staphylococcus aureus*, *Enterobacter* and *Citrobacter* were also associated with culture positive UTI in one patient each. Mixed growth was seen in one case that was also managed with oral antibiotic therapy.

 When resident/commensal organism on urethral swab was correlated with organism on post-procedural cultures, the same organism was grown only in 8 (28.5%) patients. *E Coli* was the most common resident/commensal causing UTI in 6 (21.4%) patients. Also, *Klebsiella* and *Staphylococcus Aureus* caused UTI in one (3.5%) patient each. The propensity of *E coli* as a commensal to cause UTI in our study was 6/13(46%). Also, episode of febrile UTI in these patients was 3 (23%). Interestingly, all eight patients in which the same commensal was the cause of UTI, were part of the control group. These results indicate that whenever pathogenic resident/commensal bacteria are present in urethra they may have some role to play in post-procedural UTI’s. Considering only gram negative bacteria as uro-pathogenic, the rate of UTI in the control group (n = 96) was found to be significantly more in those that grew uro-pathogenic bacteria [47.06% (8/17), 95% confidence interval: 23.37–70.82] versus those that did not [16.46% (13/79), 95% confidence interval: 8.28–24.63] at p = 0.006 using Chi square test.

### Time to occurence of UTI after procedure

Post-procedure urine cultures were taken at 2, 7 and 30 days. Total of 28 patients presented with UTI and 6 (21.4%), 17 (60.7%) and 5 (17.8%) were diagnosed on day 2, 7 and 30 post-procedure urine culture samples respectively (Table 4).

**Table 4:** Table showing time to occurrence of UTI.

TIME TO OCCURENCE OF UTI POST PROCEDURE			
DAYS POST PROCEDURE	CONTROL GROUP	INTERVENTION GROUP	TOTAL
DAY 2	5	1	6
DAY 7	12	5	17
DAY 30	4	1	5
TOTAL	21	7	28

Incidentally, no patient presented with sepsis, prostatitis, epididymo-orchitis or pyelonephritis. None of the patients required intravenous antibiotic therapy or hospital admission. All twenty-eight patients with diagnosed UTI were given a 3-day course of empirical antibiotic treatment which was later changed according to drug sensitivity report if clinically non-responsive. Repeat urine routine and culture was done following completion of course of antibiotics or at day 7 and 30 as per protocol. No further antibiotics were given if symptoms resolved and urine culture was sterile. Side effects of povidone-iodine like local inflammatory reaction, irritation, metabolic abnormality were not seen in any of the patients in the intervention group.

## Discussion

UTI by definition is an inflammatory response of the urothelium to bacterial invasion that is usually associated with bacteriuria and pyuria. Studies have indicated that bacteria may reside on the urothelium in the absence of significant bacteriuria^9,10^. The distal urethral commensal profile in our study compares well with other studies in literature^10,11^. In our study, common uro-pathogens like *E Coli*, *Klebsiella*, *Morganella*, *Proteus*, *Pseudomonas*, *Streptococcal* species and *Enterococci* could also be demonstrated as resident flora in 65 (33.9%) cases. The significance of this distal urethral resident flora in causing UTI is not well understood.

Several prospective trials of patients with pre-procedure sterile urine, report culture-proven UTI rate of 1 to 21% after cystoscopy without antimicrobial prophylaxis^12-16^. A higher baseline UTI rate in the control group of our study may be related to use of insignificant bacteriuria (<10^5^cfu/ml) as inclusion criteria; rigid cystoscopy as against flexible cystoscopy; our definition of UTI including all significant (>10^5^ cfu/ml) growths on post-cystoscopy cultures; and, non-use of prophylactic antibiotic in any case. Local antiseptic protocols could also have some bearing to this effect. The guidelines recommend to give antibiotic prophylaxis only when aberrant host factors could increase the probability or significance of infection like advancing age, poor nutrition, anatomic abnormalities, immunodeficiency, etc^2^. Previous history of UTI and a bacterial growth less than 10^5^ cfu could also predispose to UTI but they were not considered as decision making factors in this study. Nonetheless, there lies a risk of UTI in healthy culture-negative patients pointing its aetiology towards either urethral resident/commensals or inoculation through asepsis during the procedure.

Studies on bladder irrigation with povidone-iodine (2%) in the prevention of UTI’s after single or intermittent urethral catheterization have shown a significant decrease in bacteriuria and UTI^17,18^. As a corollary, irrigating distal urethra with 2% povidone-iodine solution could be one way of decreasing the incidence of UTI in culture-negative males, undergoing cystoscopy. To the best of our knowledge, this issue has not been addressed in any study till date. We observed a significant decline in UTI rate from 22% in the control group to 7.2% in the intervention group that received distal urethral irrigation with 2% povidone-iodine. Similarly, the incidence of febrile UTI was more in control group than intervention group. Importantly, there was neither any difficulty in visualizing urethral or bladder mucosa after povidone-iodine irrigation, nor any residual urethral irritation or pain in any patient. An aqueous solution of povidone-iodine is easily washed and rinsed away through irrigation without leaving any stain or lasting effects on the mucosa.

Among the patients who suffered UTI, only eight patients showed the same organism in pre-procedure urethral swab and post-procedure urine culture. That means the urethral swab could correctly identify post-procedural significant bacteriuria/UTI in only 8/28 cases even with special culture methodology. In other words, a direct causative analogy could be suggested only in these eight patients. While this percentage is not sufficient enough to recommend routine use of specialized urethral culture swab before all cystoscopies, the fact that all these 8 patients were from the control group supports the fact that irrigation with povidone-iodine solution helped in decreasing UTI and antibiotic intake.

Despite the fact that most predominant bacteria grown were staphylococci or enterococci, they were identified as causative organisms on urine culture in one odd patient, that too when they were not the predominant growth on pre-op urethral swab. However, Staphylococci are well known to cause nosocomial and opportunistic infections. And patients with compromised immune systems may be at risk of developing infection^19-21^. Therefore, including urethral irrigation with povidone-iodine as a standard surgical procedure may be even more important in immuno-compromised patients, but remains to be studied.

The most common organisms causing UTI in our series were *E Coli* followed by *Klebsiella* and *Pseudomonas*, together accounting for 86% cases. This indicates the propensity of these uro-pathogenic bacteria to cause UTI, commiserate with existing literature^22^. The propensity of *E coli* to cause UTI was the highest, among all bacteria which were demonstrated on urethral swab. We also found a significantly higher UTI rate (47.06 vs 16.46%, p = 0.006) whenever gram negative enterobacteriaceae were grown on swab culture and no povidone-iodine instillation was used.

Most (82.1%) of the patients with UTI presented within 7 days of the procedure. It further explains that after exposure to the pathogen during the office cystoscopy, whether exogenous microorganism or the resident flora, most of the patients become symptomatic within a week.

Limitations of our study include the use of a rigid cystoscope for office cystoscopy, which may be more traumatic and hence more prone to causing deeper inoculation of bacteria compared to flexible cystoscopy which is often practised in resource rich hospitals. However, worldwide rigid cystoscopy remains the most prevalent cystoscopy type, and is likely to remain so in the near future. Therefore to that extent, the study findings are more generalizable to a wider population. Moreover, resident florae include many types of bacteria that are otherwise difficult to demonstrate using routine culture methods using standardized reading criteria. With our method of urethral swab culture in the study, we identified only the most prevalent growth and therefore may have missed UTI’s caused by less prevalent bacteria. This may have reduced the correlation of pre and post procedure cultures. Overall, a high prevalence of uro-pathogenic bacteria as resident flora in distal urethra of males, and reduction of UTI’s with use of povidone-iodine irrigation suggests that urethral irrigation with povidone-iodine should be done in all males undergoing office cystoscopy. A similar role of povidone-iodine irrigation of urethra before urethral catheterization in the operation theatres or hospital wards or intensive care units to reduce catheter-associated urinary tract infections remains to be studied.

## Conclusion

Distal urethral resident/commensal flora is diverse, often harbouring known uro-pathogens and has got some role in causing UTI after office cystoscopy in men. Our urethral swab technique could correctly identify causative organism in only 8/28 cases even with special culture methodology. Irrespective of urethral swab culture, irrigating distal urethra with povidone-iodine seems effective in decreasing post-procedural UTI. We recommend routine inclusion of aqueous 2% povidone-iodine urethral irrigationas part preparation before any office cystoscopy. This may help reduce the use of antibiotics and improve the cost of treatment.

(Trial Registration number: National Trial Registry, India(CTRI/2019/08/020623) [Registered on 07/08/2019], Full trial protocol can be accessed from the journal’s website).

## Data Availability

The entire data set of the study is available with the authors for review for any scientific purpose, as and when necessary.
